# Does Spontaneous Echo Contrast in the Left Atrial Appendage Increase Thromboembolism Risk After Left Atrial Appendage Closure? A Retrospective Study on Its Impact on Device-Related Thrombosis and Arterial Thromboembolic Events

**DOI:** 10.1155/cdr/1849432

**Published:** 2025-04-11

**Authors:** Kandi Zhang, Hanwen Yu, Yihua Lu, Peng Zhang, Dongsheng Liu, Jianpeng Huang, Jing Zhou, Yiqian Yuan, Zongqi Zhang, Qingyong Zhang, Qing He, Junfeng Zhang

**Affiliations:** ^1^Department of Cardiology, Shanghai Ninth People's Hospital, Shanghai Jiao Tong University School of Medicine, Shanghai, China; ^2^Shanghai Baoshan District Yanghang Town Community Health Service Center, Shanghai, China

**Keywords:** atrial fibrillation, device-related thrombosis, left atrial appendage closure, spontaneous echo contrast, thromboembolism

## Abstract

**Background:** Left atrial appendage closure (LAAC) prevents arterial thromboembolic events (ATEs) in nonvalvular atrial fibrillation (AF). Spontaneous echo contrast (SEC) is an independent risk factor for left atrial appendage (LAA) thrombosis; however, there is little knowledge about the relationship between preoperative SEC and the increased risk of device-related thrombosis (DRT) or ATEs in patients with AF who have undergone LAAC.

**Methods:** This retrospective study focused on patients with nonvalvular AF who successfully underwent LAAC surgery. Transesophageal echocardiography (TEE) was used to assess preoperative LAA status. SEC in LAA Grades 0–2 was defined as LAASEC−, and Grades 3–4 or previously diagnosed LAA thrombus formation as LAASEC+.

**Results:** A total of 519 AF patients (432 in LAASEC− group and 87 in LAASEC+ group) who underwent LAAC were included. At the 1-year follow-up, there was no significant difference in the incidence of DRT (2.5 vs. 3.8%, *p* = 0.636), ATEs (0.5 vs. 0%, *p* = 0.525), and all-cause mortality (2.1% vs. 2.3%, *p* = 0.899) between the LAASEC− group and the LAASEC+ group. However, the LAASEC+ group had higher proportions of cauliflower-type LAA and ≥ 3 lobes.

**Conclusions:** The difference in preoperative LAA thrombosis or LAASEC was not related to the incidence of DRT or ATEs in AF patients within 1 year after LAAC.

## 1. Introduction

Atrial fibrillation (AF) is a common type of clinical tachyarrhythmia, with the incidence rate in adults worldwide estimated to be approximately 2%–4% [1]. Arterial thromboembolic events (ATEs) are the leading cause of disability and mortality in nonvalvular AF. AF accounts for approximately 15%–25% of ischemic stroke events [2]. Real-world exploration suggests that > 90% of atrial thrombus in patients with AF originates from the left atrial appendage (LAA) [3]. Left atrial appendage closure (LAAC) is an alternative to lifelong oral anticoagulants for patients with AF, reducing the risk of bleeding caused by long-term oral anticoagulants while decreasing the risk of ischemic stroke [4].

Patients with AF will receive antithrombotic treatment for some time, following implantation with a LAA occlusion device, to prevent device-related thrombosis (DRT). The incidence of DRT is low but associated with a 4–5-fold increased risk of ischemic events [5]. Studies have also shown a significant correlation between DRT and an increased risk of composite endpoint events, including death, ischemic stroke, and systemic embolism [6]. DRT mainly occurs in the early stages after the implantation of a LAA occlusion device; its principle is the incomplete endothelialization of the occluder surface. The American Society for Cardiovascular Imaging and Intervention suggested that transesophageal echocardiography (TEE) or cardiac computerized tomography (CT) should be conducted 45–90 days after LAAC, to evaluate the occurrence of leakage around the occluder and the DRT [7].

Spontaneous echo contrast (SEC) in the LAA is an abnormal ultrasound finding caused by stasis observed during TEE, a prethrombotic state. Left atrial appendage spontaneous echo contrast (LAASEC) and LAA thrombosis are independent risk factors for stroke events in patients with AF [8, 9]. The more significant the LAASEC, the higher the risk of LAA thrombosis and thrombus detachment, causing ATEs in patients with AF. In theory, patients with AF at high risk of ischemic stroke should benefit from LAAC therapy. However, we suspect that patients with high LAASEC levels before LAAC are prone to thrombus formation in the left atrium, and the incidence of postoperative DRT and ATEs may increase. Therefore, in this study, we aimed to compare the effects of different LAASEC on the incidence of DRT and ATEs within 12 months after LAAC in patients with AF.

## 2. Materials and Methods

### 2.1. Study Population

In this retrospective study, we investigated patients with nonvalvular AF, who underwent LAAC and completed TEE at Shanghai Ninth People's Hospital, Shanghai Jiao Tong University School of Medicine between January 2019 and January 2023, with a follow-up period of 12 months. Indications and contraindications for LAAC were based on expert consensus [7]. In order to minimize the potential impact of missing anticoagulant therapy on preoperative LAASEC, only patients who received at least 2 weeks of standard-dose anticoagulant therapy before LAAC were included in the study. All patients underwent left atrial CT angiography and TEE within 3 days before surgery to exclude thrombi and evaluate the LAA structure. Patients who met the following criteria were not excluded from the study: those previously diagnosed with thrombus formation in the LAA on TEE, had undergone additional anticoagulation therapy, had their thrombus excluded on TEE follow-up, and ultimately successfully underwent LAAC. The exclusion criteria were valvular heart disease, history of valve replacement or valve replacement surgery, complex congenital heart disease, recent ischemic stroke, deep vein thrombosis, pulmonary embolism, active inflammatory disease, severe liver or kidney dysfunction, and failure to adhere to standardized postoperative antithrombotic treatment.

### 2.2. Data Collection and Assessments

The clinical characteristics of the subjects were retrieved from the electronic medical record system of Shanghai Ninth People's Hospital, Shanghai Jiao Tong University School of Medicine, and it included the following data: demography (age, gender, and body mass index); medical history (congestive heart failure, hypertension, diabetes, previous stroke/transient ischemic attack (TIA), vascular disease, coronary artery disease, and antithrombotic drug program after LAAC); biochemical parameters (brain natriuretic peptide and estimated glomerular filtration rate); and echocardiographic parameters (diastolic left atrial diameter and left ventricular ejection fraction). Diagnosis of AF was determined by the patient's medical history and Standard 12 lead and/or dynamic electrocardiogram monitoring. The definition and classification of AF were from published guidelines [1].

### 2.3. TEE

**Figure 1:**
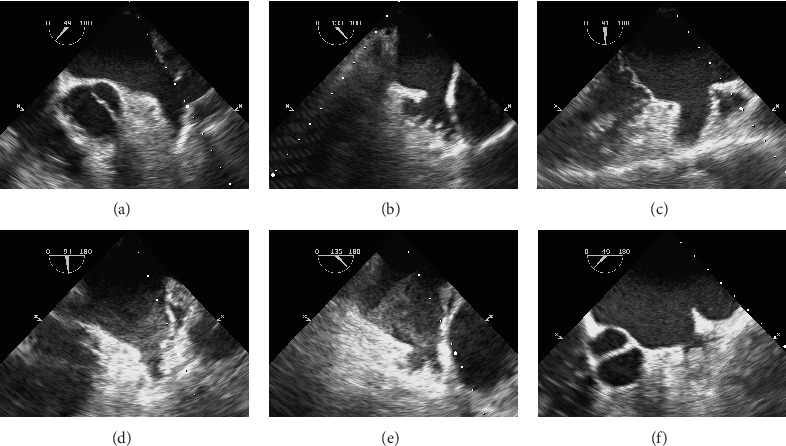
TEE images of LAASEC at different levels. (a–e) LAASEC Grades 0–4 in sequence. (f) Thrombus formation in the LAA.

Before TEE, the patient was required to fast for 6 h and receive local pharyngeal anesthesia. TEE was performed using a Philips X7-2t transesophageal ultrasound probe inserted into the esophagus at 25–35 cm depth (Philips, Netherlands). The LAA was inspected on different fault planes ranging from 0° to 180° to detect the presence of LAASEC. LAASEC is the appearance of dynamic “smoke-like” echoes in LAA, with rotational motion characteristics that cannot be eliminated by changing the gain setting [10]. According to the differences in TEE, LAASEC was divided into five levels [11]. Patients with LAASEC Grades 3–4 and those previously diagnosed with LAA thrombus by TEE were included in the LAASEC+ group, while the LAASEC− group consisted of patients with LAASEC Grades 0–2 (Figure 1). Experienced echocardiographic physicians performed all TEE; the results were independently evaluated by two echocardiography physicians blinded to the study protocol. Referring to the imaging characteristics of DRT proposed in the PROTECT-AF study [12], the standards for diagnosing DRT by TEE are as follows: (i) high-density echo on the left atrial side of the device; (ii) imaging artifacts that cannot be explained; (iii) inconsistency with the normal healing or device imaging appearance; (iv) visibility in multiple planes on TEE; and (v) connection with the device.

The standards for diagnosing DRT by CT imaging: The characteristic manifestation of DRT on CT is the presence of contrast agent filling defects on the atrial side of the occluder.

### 2.4. LAAC

Indications, contraindications, preoperative preparation, and surgical processes for LAAC were determined by the SCAI/HRS Expert Consensus Statement on transcatheter LAAC [7]. Indications for LAAC: For male patients with nonvalvular AF and a CHA2DS2-VASc score of ≥ 2 or female patients with a CHA2DS2-VASc score of ≥ 3, who also have one of the following conditions: (i) Those who are unsuitable for long-term anticoagulant therapy, including patients who are unwilling or unable to take warfarin and nonvitamin K antagonist oral anticoagulants (NOACs) for special reasons; (ii) patients who have had a stroke or embolic event despite long-term standardized anticoagulant therapy; (iii) patients at high risk of bleeding, including those with a HAS-BLED score of ≥ 3 or those who require long-term use of antiplatelet drugs. Contraindications for LAAC: (i) left atrial diameter > 65 mm, left atrial thrombosis detected by TEE, severe mitral valve disease, or pericardial effusion > 3 mm; (ii) life expectancy < 1 year; (iii) low stroke risk (CHA2DS2-VASc score of 0 or 1) or low bleeding risk (HAS-BLED score < 3); (iv) other conditions requiring warfarin anticoagulation therapy in addition to AF; (v) presence of a patent foramen ovale with atrial septal aneurysm and right-to-left shunt; and (vi) patients requiring elective cardiac surgery. An independent surgeon with extensive experience performed the LAAC. Patients underwent TEE before the surgery. TEE and left atrial CT examinations were conducted 90 ± 15 days after LAAC. All surgeries were performed under intraoperative TEE guidance.

### 2.5. Anticoagulant and Antiplatelet Treatment After LAAC

After LAAC, patients received standardized antithrombotic treatment. Antiplatelet drugs include aspirin (100 mg qd), ticagrelor (90 mg bid), and clopidogrel (75 mg qd). NOAC only includes rivaroxaban (15 mg qd). The dual antiplatelet therapy (APT) (DAPT) regimen consists of aspirin combined with a P2Y12 inhibitor. The antithrombotic regimen within 90 days after LAAC mainly follows the following principles: (i) If there are no special circumstances, priority should be given to NOAC. (ii) For patients with LACbes occluders implanted and requiring long-term APT for cardiovascular and cerebrovascular diseases, DAPT should be prioritized over NOAC. (iii) For patients with Watchman occluders implanted who have undergone coronary or cerebrovascular intervention within the past 12 months, NOAC combined with DAPT or NOAC combined with single antiplatelet therapy (SAPT) may be considered. The specific decision was jointly made by the surgeon and the attending physician based on the patient's ischemia and bleeding risk assessment. (iv) For patients who cannot tolerate NOAC due to renal insufficiency or other reasons, warfarin should be used, and the international normalized ratio (INR) should be controlled at 2.0–3.0. TEE was done within 90 ± 15 days after LAAC. If TEE showed no atrial thrombus, switching to or continuing (if DAPT was originally received after surgery) the DAPT regimen and switching to long-term oral administration of SAPT 6 months after LAAC would be effected.

### 2.6. Statistical Analysis

Continuous variables with a normal distribution were represented by mean ± standard deviation. Nonnormally distributed variables were described by median and interquartile ranges; while categorical variables were expressed as frequencies and percentages. The *t*-test or Mann–Whitney *U* test was used to compare continuous variables between groups. Pearson's chi-square test was used to compare categorical variables, and Fisher's–exact test was used for situations with low event incidence. All statistical analyses were conducted using IBM SPSS Statistics Version 25. *p* < 0.05 was statistically significant.

### 2.7. Follow-up

Within 12 months after LAAC, patients will be followed up once every 2 months through telephone or outpatient visits. ATEs including ischemic stroke, TIA, acute myocardial infarction (AMI), death, and adverse events in patients within 12 months after surgery were recorded, with left atrial CT and TEE data. All patients underwent TEE 3 months after surgery and at least one left atrial CT examination between 6 and 12 months after surgery. The CT imaging features of DRT are as follows: (i) the presence of layered hypoattenuated thickening on the left atrial side of the occluder with a thickness greater than 3 mm; (ii) the left atrial side of the occluder has a pedicle or is irregular. Patients suspected of having DRT from the left atrial CT results had TEE to confirm the diagnosis. During this period, all the participants received standardized antithrombotic treatment.

## 3. Results

A total of 519 patients with nonvalvular AF (42.0% female) who successfully received LAAC were enrolled in this study, with an average age of 73.40 ± 8.13 years. The average CHA2DS2-VASc score was 3.74 ± 1.50. Among them, there were 432 and 87 cases (83.23 and 16.76%), respectively, in the LAASEC− and LAASEC+ groups. The characteristics of LAASEC− and LAASEC+ patients are shown in Table 1. There were no statistically significant differences between the two groups in sex, BMI, HAS-BLED score, occluder type, ablation history, coronary heart disease, hypertension, diabetes, liver dysfunction, renal dysfunction, previous bleeding history (Table 1), or the use of antithrombotic drugs after surgery (Table 2).

Compared to the LAASEC− group, patients in the LAASEC+ group were older (72.98 ± 8.03 vs. 75.55 ± 8.37, *p* < 0.05), had a higher proportion of persistent AF (42.1 vs. 71.3%, *p* < 0.05), a higher CHA2DS2-VASc score (3.62 ± 1.43 vs. 4.36 ± 1.70, *p* < 0.05), a larger left atrial diameter (42.76 ± 6.10 vs. 47.53 ± 6.87, *p* < 0.05), a lower LVEF (58.24 ± 7.50 vs. 55.60 ± 8.06, *p* < 0.05), a higher incidence of stroke (23.9 vs. 36.8%, *p* < 0.05), and chronic heart failure (27.5 vs. 41.4%, *p* < 0.05).

All patients successfully underwent the implantation of the LAA occluder, which was anchored during the operation. A residual shunt greater than 5 mm at the occluder edge was not detected after its release. In 519 LAAC, two types of occluders: plug and disc, were used, with 251 LACbes (Push Medical, Shanghai, China) and 268 WATCHMANs (Boston Scientific, Massachusetts, United States) implanted.

Among the preoperative LAASEC− and LAASEC+ groups, 11 cases (2.5%) and three cases (3.4%), respectively, were detected with DRT by TEE within 1 year follow-up after LAAC (Table 3), and there was no statistical significance between the two groups (*p* = 0.636). There were two cases (0.5%) of ATEs in the LAASEC− group and no ATEs in the LAASEC+ group (0%). Preoperative LAASEC was not associated with the incidence of ATEs within 1 year after LAAC (*p* = 0.525). The 1-year all-cause mortality rate in the LAASEC− and LAASEC+ groups was nine and two cases (2.1 and 2.3%), respectively. There was no statistical significance between the two groups (*p* = 0.899). At the end of the follow-up period, the proportion of antithrombotic treatment regimens between the two groups was not statistically significant (Table 2).

In addition, the morphology of the LAA differed between the two groups. In the LAASEC+ group, a higher proportion of the LAA exhibited cactus and cauliflower morphologies, with a greater frequency of 3 and ≥ 4 lobes, compared with the LAASEC− group (Table 4).

## 4. Discussion

Patients with AF experience stasis of blood flow in the atrium (especially in the LAA) due to the loss of effective contraction and relaxation of the atrium. After the accumulation of red blood cells and the blood overlaps, it appears as a “cloud-like” spontaneous enhancement on an ultrasound scan. SEC is an imaging feature of the prethrombotic state. In recent years, SEC in the LAA has been widely studied and diagnosed using TEE. According to relevant clinical studies and literature reports, LAASEC has been recognized as a precondition and independent risk factor for LAA thrombosis [13, 14]. Despite standard anticoagulant therapy, LAA thrombosis or LAASEC is associated with a high incidence of major adverse cardiovascular and cerebrovascular events [15]. Research has shown that high CHA2DS2-VASc scores, low LVEF, and LAASEC are risk factors for left atrial thrombosis [16]. Patients with chronic AF, those who did not receive anticoagulant therapy, or those who received warfarin anticoagulant treatment but failed to meet the INR monitoring standards were more likely to develop left atrial thrombosis or LAASEC [17].

In the management of AF, preventing the occurrence of embolic events has always been the focus of treatment. LAAC seals the LAA, thereby blocking the channels for thrombus formation and detachment and effectively reducing the incidence of embolic events. The effectiveness of LAAC has been recognized in clinical guidelines. Multiple randomized controlled trials and observational studies have shown that LAAC is more effective than oral anticoagulants in preventing thrombotic events caused by AF, while significantly reducing the risk of bleeding [18, 19]. Therefore, LAAC is a good choice for avoiding embolic events in patients with AF; however, it is not without risks. DRT is a common complication of LAAC. There are many triggering factors, which may include a high CHA2DS2-VASc score, TIA/stroke history, permanent AF, vascular disease, large LAA, and low LVEF [5]. In addition, selecting implanted instruments and postoperative antithrombotic drugs may play an important role [6]. According to single-center data from the Helmut-G Center in Germany, the incidence of DRT was 4.49% [20], contrary to a multicenter study in France that showed that DRT reached 7.2% [21]. A multicenter registration study [22] found that the median time for the first detection of DRT events after LAAC surgery was 93 days, and 17.9% of patients were diagnosed with DRT 6 months after surgery. Through strengthening or changing anticoagulation strategies, DRT was eliminated in 79.5% of the patients. Two years of follow-up showed that patients who underwent DRT had a higher mortality and ischemic stroke incidence rate (20% and 13.8%), respectively. DRT may increase the risk of thrombotic events after LAAC, thereby reducing the net clinical benefits of surgery. Consequently, reducing the incidence of DRT and improving the safety of postoperative cardiovascular and cerebrovascular diseases are problems that require further research.

The possible risk factors for DRT were partially the same as those associated with LAASEC. However, there is limited research on whether patients with AF with high preoperative LAASEC levels are at an increased risk of DRT after undergoing LAAC. The EWOLUTION study [23] found that the occurrence of DRT in patients with AF after LAAC using the WATCHMAN occluder was associated with nonparoxysmal AF, LAA opening diameter, and SEC. Therefore, more clinical studies are required to clarify the relationship between preoperative LAASEC levels and the risks of DRT and ATEs after LAAC to better guide clinical practice.

Our study found that the degree of preoperative LAASEC did not affect the incidence of DRT, ATEs, or all-cause mortality within 1 year after LAAC. However, many predictive factors for DRT were significantly more common in the LAASEC+ group. First, it indicates that LAAC is equally effective in preventing ischemic stroke in patients with AF with a high risk of LAA thrombus. Patients with preoperative LAASEC+ or previous thrombus formation in the LAA are not too bothered about the effectiveness of LAAC. Second, the mechanism of preoperative thrombus formation in the LAA differs from that of postoperative DRT.

We compared the similarities and differences in the morphology and structure of the LAA between the LAASEC− and LAASEC+ groups. In the LAASEC+ group, the proportion of cauliflower-shaped LAA was significantly higher, consistent with the findings of Di Biase et al. [24]. Previous studies have confirmed that cauliflower-shaped LAA is an independent risk factor for thrombosis in AF and stroke. In addition, He et al. [25] suggested that the more complex and lobulated the internal structure of the LAA, the easier it is for thrombosis to occur. In this study, the proportion of patients in the LAASEC+ group with 3 or ≥ 4 lobes in the LAA was markedly higher than that of the LAASEC− group. This could be due to the increased number of lobes in the LAA, which redirected blood from the left atrium to the LAA, leading to reduced blood flow velocity and facilitating thrombus formation. Therefore, we deduced that the morphology and number of the LAA lobes are important factors affecting LAASEC. However, the LAAC isolates the LAA from the left atrium, making it an ineffective cavity without blood flow after surgery, thereby shielding the influence of the shape and number of lobes of the LAA. Despite the existence of systemic risk factors (age, CHA2DS2-VASc score, type of AF, etc.), the influence of local risk factors (atrial appendage structure) was eliminated, explaining why there was no difference in the incidences of DRT and ATEs among the different LAASEC groups after LAAC.

Following an increasing number of published clinical studies (EWOLUTION/PINNACLE FLX/PRAGUE-17) [26–28], the 2023 ACC/AHA/ACCP/HRS guidelines for the Diagnosis and Management of AF [29] have elevated the recommendation level of LAAC from Class IIb (2019) [30] to IIa for medium- to high-risk patients with embolism in AF with long-term oral anticoagulation (OAC) contraindications. New research [31] suggests that imaging results are superior to CHA2DS2-VASc scores in evaluating the risk of embolism in AF. We anticipate that soon, preoperative LAASEC results will become the primary reason for stratifying the risk of embolism in patients with AF. LAAC is as effective in preventing embolic events in high-risk populations with high levels of LAASEC and offers greater benefits to patients. It may replace long-term oral anticoagulants and become a Class I recommendation for this subgroup.

## 5. Conclusion

Although AF patients with higher preoperative LAASEC levels have clinical features such as older age, a higher proportion of persistent AF, higher CHA2DS2-VASc scores, larger left atrial diameter, more lobes of the LAA, lower LVEF, and a higher incidence of previous and chronic heart failure, the incidence of DRT or stroke after LAAC does not increase. This confirms that the benefits of LAAC for AF patients are not affected by preoperative LAASEC, which may be related to LAAC eliminating the impact of LAA structural differences on thrombus formation.

### 5.1. Limitations

This study was a retrospective analysis involving two different types of LAA occluders. No statistically significant difference was found in the use of the two different occluders in the LAASEC− and LAASEC+ groups; however, the various types and sizes of occluders may have an impact on the rate of endothelialization on the occluder surface after LAAC. Additionally, the size of the LAA opening and volume are crucial for the selection of an intraoperative occluder and are closely related to preoperative SEC. However, these factors were not included in the study and may have impacted the research results. Although preoperative standardized anticoagulant therapy may be beneficial in reducing the risk of preoperative LAA thrombosis and improving the success rate of LAAC, it may also lower the preoperative LAASEC level, resulting in a lower proportion of patients in the LAASEC+ group. The heterogeneity of postoperative antithrombotic treatment regimens received by patients in the study may affect the risk assessment of DRT and ATEs. Although there was no significant difference in the proportion of various postoperative antithrombotic regimens between the two groups, different antithrombotic regimens may potentially influence the observed research outcomes. Previous studies have demonstrated that there is no significant difference in thrombotic events between OAC and APT following LAAC surgery, yet OAC reduces the proportion of DRT. Even after excluding patients receiving SAPT, OAC still offers benefits compared to patients receiving DAPT [32]. In the EWOLUTION study, the therapeutic effects of NOAC, warfarin, and DAPT appear comparable [33]. The follow-up period was limited to 1 year, so long-term clinical events beyond this timeframe could not be evaluated. Due to the limited number of events observed in the study, the statistical analysis' capability is constrained, potentially impacting the significance of the results. The small sample size could result in potential selection bias and variability in the results, thereby complicating the detection of actual but weak associations, particularly in retrospective studies. Future studies should consider larger sample sizes and longer follow-up periods to enhance the robustness of research results.

## Figures and Tables

**Table 1:** The baseline of the characteristics of the patients.

	**LAASEC− (** **n** = 432**)**	**LAASEC+ (** **n** = 87**)**	**p**
Age, years	72.98 ± 8.03	75.55 ± 8.37	0.007
Female, *n* (%)	180 (41.7%)	38 (43.7%)	0.729
BMI (kg/m^2^)	24.90 ± 3.65	25.36 ± 3.36	0.282
CHA2DS2-VASc	3.62 ± 1.43	4.36 ± 1.70	< 0.001
Has-BLED	2.48 ± 1.05	2.69 ± 1.05	0.089
LVEF (%)	58.24 ± 7.50	55.60 ± 8.06	0.003
Left atrial diameter (mm)	42.76 ± 6.10	47.53 ± 6.87	< 0.001
Disc-type occluder, *n* (%)	205 (47.5%)	46 (52.9%)	0.356
History of ablation, *n* (%)	91 (21.1%)	11 (12.6%)	0.071
Non-PAF, *n* (%)	182 (42.1%)	62 (71.3%)	< 0.001
Coronary disease, *n* (%)	153 (35.4%)	29 (33.3%)	0.71
Congestive heart failure, *n* (%)	119 (27.5%)	36 (41.4%)	0.01
Hypertension, *n* (%)	323 (74.8%)	69 (79.3%)	0.369
Diabetes, *n* (%)	124 (28.7%)	34 (39.1%)	0.055
Prior stroke, *n* (%)	103 (23.9%)	32 (36.8%)	0.013
Chronic kidney disease, *n* (%)	88 (20.4%)	20 (23.0%)	0.583
EGFR (mL/min/1.73m^2^)	72.86 ± 22.47	70.00 ± 23.72	0.284
Liver dysfunction, *n* (%)	11 (2.5%)	2 (2.3%)	0.893
History of bleeding, *n* (%)	29 (6.7%)	8 (9.2%)	0.412

Abbreviations: BMI, body mass index; eGFR, estimated glomerular filtration rate; LVEF, left ventricular ejection fraction; non-PAF, nonparoxysmal atrial fibrillation.

**Table 2:** Postoperative antithrombotic treatment.

	**LAASEC− (** **n** = 432**)**	**LAASEC+ (** **n** = 87**)**	**p**
Antithrombotic drugs			0.388
DAPT, *n* (%)	52 (12.0%)	14 (16.1%)	
NOAC, *n* (%)	310 (71.8%)	62 (71.3%)	
NOAC+DAPT, *n* (%)	7 (1.6%)	0 (0%)	
NOAC+SAPT, *n* (%)	59 (13.7%)	9 (10.3%)	
Warfarin, *n* (%)	4 (0.9%)	2 (2.3%)	

Abbreviations: DAPT, dual antiplatelet therapy; NOAC, new oral anticoagulants; SAPT, single antiplatelet therapy.

**Table 3:** Adverse events.

	**LAASEC− (** **n** = 432**)**	**LAASEC+ (** **n** = 87**)**	**p**
DRT, *n* (%)	11 (2.5%)	3 (3.4%)	0.636
ATEs, *n* (%)	2 (0.5%)	0 (0.0%)	0.525
All-cause death, *n* (%)	9 (2.1%)	2 (2.3%)	0.899

Abbreviations: ATEs, arterial thromboembolic events; DRT, device-related thrombus.

**Table 4:** Left atrial appendage morphology.

	**LAASEC− (** **n** = 432**)**	**LAASEC+ (** **n** = 87**)**	**p**
Types, *n* (%)			0.002
Chicken wing	212 (49.1%)	32 (36.8%)	
Windsock	134 (31.0%)	23 (26.4%)	
Cactus	63 (14.6%)	19 (21.8%)	
Cauliflower	23 (5.3%)	13 (14.9%)	
Lobes, *n* (%)			0.002
1 lobe	65 (15.1%)	12 (13.8%)	
2 lobes	207 (47.9%)	24 (27.6%)	
3 lobes	125 (28.9%)	39 (44.8%)	
≥ 4 lobes	35 (8.1%)	12 (13.8%)	

## Data Availability

Data are available upon reasonable request (contact the corresponding author Dr. Junfeng Zhang).
